# MiR-346 suppresses cell proliferation through SMYD3 dependent approach in hepatocellular carcinoma

**DOI:** 10.18632/oncotarget.18060

**Published:** 2017-05-22

**Authors:** Weiyou Zhu, Jing Qian, Ling Ma, Pei Ma, Fengming Yang, Yongqian Shu

**Affiliations:** ^1^ Department of Oncology, The First Affiliated Hospital of Nanjing Medical University, Nanjing, Jiangsu Province, P.R. China; ^2^ Oncology Center, Shengze Hospital of Nanjing Medical University, Wujiang, Jiangsu Province, P.R. China

**Keywords:** miRNA, proliferation, untranslated regions, hepatocellular carcinoma, SMYD3

## Abstract

**BACKGROUND & AIMS:**

The miRNAs are demonstrated to be involved in the carcinogenesis of hepatocellular carcinoma (HCC) and some exhibit potential value for oncotherapy. This study was designed to explore the role of miR-346 in the pathogenesis of hepatocellular carcinoma.

**METHODS:**

High throughput screening was employed following with Real time-PCR to investigate the candidate miRNAs. 5-ethynyl-2-deoxyuridine (EdU) assay, CCK-8, transwell assay, cell cycle assay, luciferase reporter assay, western blot and mice xenotransplantation model were performed in the present study.

**RESULTS:**

We found miR-346 was significantly down-regulated in the HCC tissues compared with the non-tumor controls and was associated with the tumor size and TNM grade. Additionally, the *in vitro* and *in vivo* assays confirmed that miR-346 suppressed the proliferation of HCC. Then, bioinformatic algorithms and luciferase reporter assays proved that miR-346 directly targeted SET and MYND domain containing 3(SMYD3). We also performed the rescue experiments by inhibiting the expression of SMYD3 and found the down-regulation of SMYD3 could neutralize the inhibitory effects of miR-346 on HCC. At last, the cox proportional hazards analysis showed that low expression of miR-346 was an an independent prognostic factor for HCC.

**CONCLUSION:**

Our findings illuminated miR-346 targeting SMYD3 to inhibit the proliferation of HCC and its down-regulation predicts a poor prognosis.

## INTRODUCTION

Hepatocellular carcinoma (HCC) has been demons-trated to be one of the most common life-threatening malignancies worldwide [1, 2]. Although multiple approach including operation therapy, chemotherapy and immunotherapy has been developed in recent decades, the prognosis of HCC still remain poor. The exact underlying mechanisms mainly lead to the development and progression of HCC still remain elusive and prevent the advances of medical therapies for the HCC patients [3].

In recent years, more attention has been paid to explore microRNA, also known as miRNAs, which were defined as short non-coding RNAs with approximately length of 19-25nt [4, 5]. These miRNAs were considered to participate in gene modulation by directly binding to the 3’-untranslated region (3’-UTR) of their target genes, leading to translation inhibition or degradation [6, 7]. Via the weakening of target mRNA, miRNA has a significant role at the post-transcriptional level and contributes to the regulation of a lot of biological processes, including cell proliferation, migration, invasion or apoptosis [6, 8-10]. Importantly, various studies have demonstrated that miRNA could act as tumor promoters or as tumor suppressors via targeting anti-oncogenes or oncogenes in HCC [11, 12].

SMYD3 was a well-known oncogene involved in human malignant tumors. Researchers has identified the residue of histone 4 lysine-5 (H4K5) as a primary chromatin target of SMYD3. Interestingly, up-regulation of SMYD3 was found in various kinds of tumors, including HCC, lung adenocarcinoma, breast cancer, and pancreatic ductal adenocarcinomas, which indicating SMYD3 might play an important role in the development of human cancer [13-16]. Furthermore, growing evidences have suggested that SMYD3 expression in mice was required for chemically induced liver or colon cancer. In these organs, the endogenous expression of SMYD3 in human liver or colon could regulate JAK/STAT3 or C-Myc associated signaling pathway resulting the abnormal of cell proliferation and epithelial-mesenchymal transition [17-19]. However, how the expression of SMYD3 is modulated in the progression of HCC is still not well known.

In our study, real-time-PCR demonstrated that miR-346 was significantly down-regulated in HCC tissues and cells compared with the non-tumorous tissues and cells. *Chi-square* analysis showed the expression levels of miR-346 were associated with tumor size and TNM grade. In addition, the results of the *in vitro* and *vivo* assays indicated miR-346 suppressed the proliferation of HCC cells. At last, we performed the Kaplan–Meier analysis and Cox proportional regression analysis and found low expression of miR-346 could be a potential biomarker for predicting the survival of HCC patients.

## RESULTS

### MiR-346 was aberrantly down-regulated in HCC tissues and cell lines

**Figure 1:**
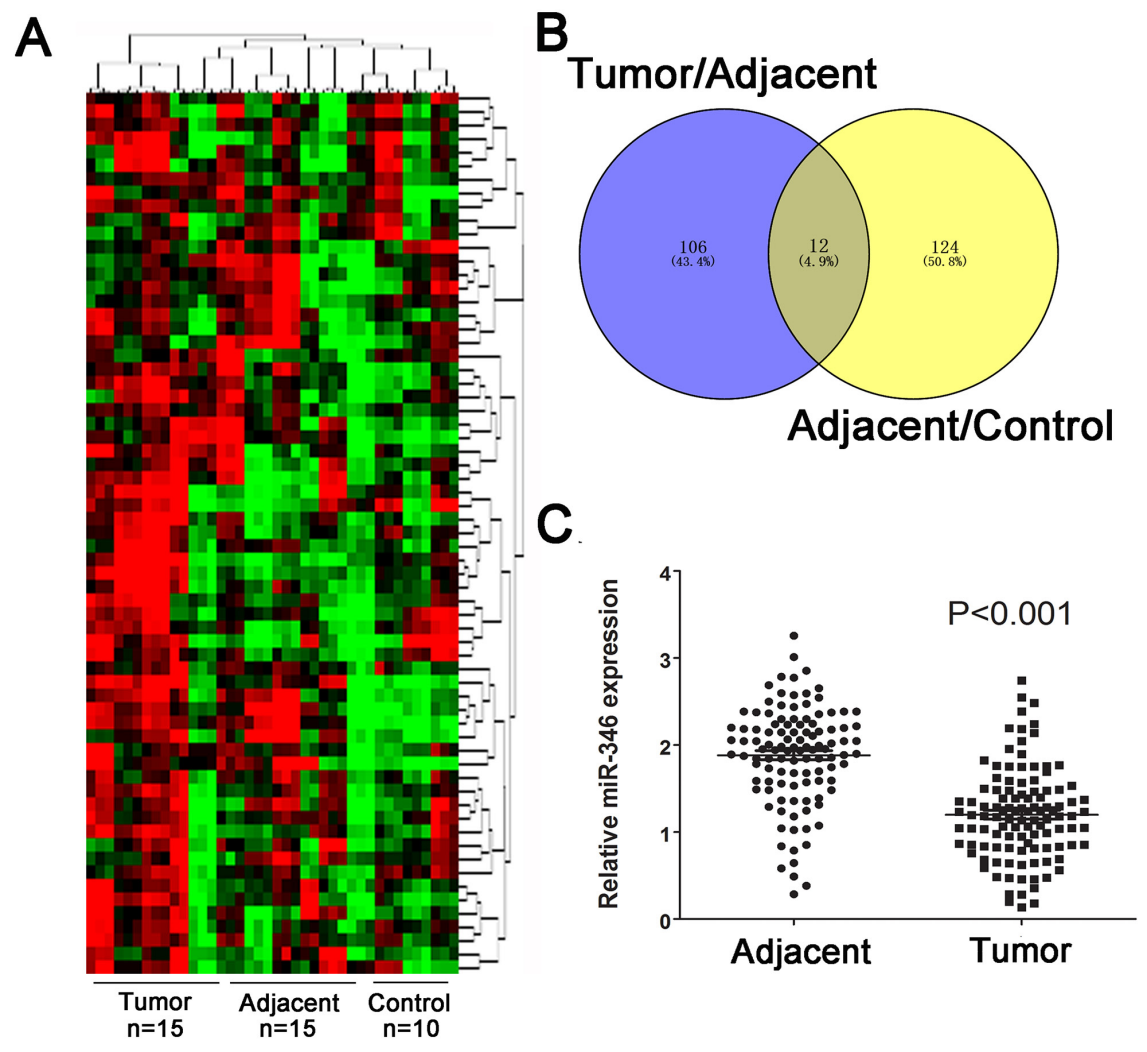
MiR-346 was aberrantly down-regulated in HCC tissues and cell lines **(A)** The cluster analysis was performed to analyzed the expression profile of miRNAs in HCC tumor tissues (n=15), adjacent tissues(n=15) and control tissues(n=10). **(B)** Venny analysis for the different expressed miRNAs. **(C)** The expression of miR-346 in 110 pairs of human HCC tissues were demonstrated to be significantly down-regulated compared with the the corresponding adjacent tissues. Data are presented as means±SEM.

**Figure 2:**
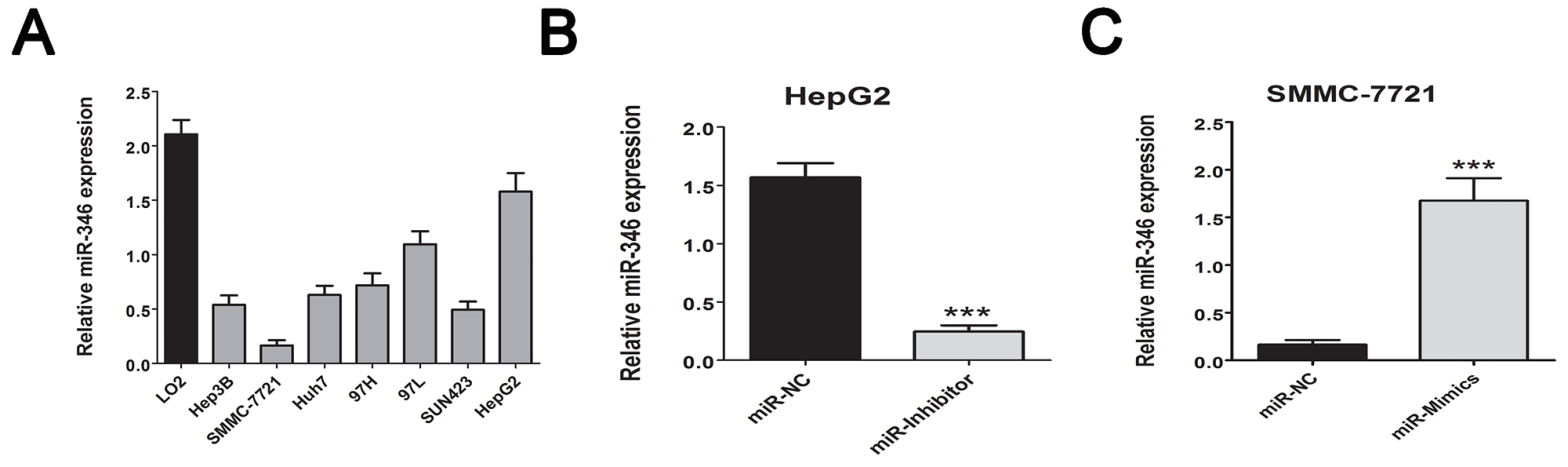
MiR-346 was aberrantly down-regulated in HCC cell lines **(A)** We confirmed the miR-346 expression was down-regulated in the HCC cell lines in comparison with the human normal L02 cells. **(B, C)** By using the transfection with miR-346 mimics and inhibitor, we respectively up-regulated the expression of miR-346 in SMMC-7721 and down-regulated the miR-346 in HepG2. We also transfected SMMC-7721 cells with the up-regulated lentivirus of miR-346 to perform further *in vivo* assay. Data are presented as means±SEM.

We firstly screening the different expression of miRNAs in human HCC tissues including 15 tumor tissues and 15 corresponding adjacent tumor tissues, the 10 healthy liver tissues obtained from patients who received hepatectomy due to benign liver diseases. As presented in Figure 1A, the abnormal miRNA expression profile was observed in the three groups. The fold change value and p value was used as filter condition. MiRNA indicated at least 2 fold change with p<0.05 will be considered as significant. The Venny analysis indicated that 12 miRNAs was screened as candidate miRNAs (Figure 1B). Among the candidate, miR-346 presented the most significant fold change and p value (Table 1). By using real-time-PCR, we found the expression of miR-346 was significantly down-regulated in 110 pairs of human HCC tissues compared with the corresponding adjacent tissues (Figure 1C). Then we examined the expression of miR-346 in human HCC cell lines and human normal liver cells, the results showed miR-346 was over-expressed in human normal L02 cells in comparison with HCC cells (Figure 2A). The aberrant expression of miR-346 indicated that miR-346 might display a crucial role in the carcinogenesis of HCC. Hence, Chi-square analyses were conducted to found the potential correlations between miR-346 and the clinical characteristics. The results of Chi-square analyses showed the expression levels of miR-346 were associated with tumor size (P=0.012) and TNM grade (P=0.006) of HCC patients (Table 2). To further explore the potential effects of miR-346, we treated SMMC-7721 with miR-346 mimics. Similarly, HepG2, which had a relative high expression of miR-346, was transfected with miR-346 inhibitor to down-regulate the miR-346 expression. The expression of miR-346 of pretreated cells was shown in Figure 2B and 2C.

**Table 1:** Candidate miRNA screened with microarray database

p Value	FC (abs)	Regulation	Gene symbol
0.0033945	9.881922	Up	miR-21
0.0014345	13.905087	Up	miR-128
0.0033921	4.211089	Up	miR-218
0.0145723	3.4214008	Up	miR-92
0.0199178	9.061603	Up	miR-1
0.0026899	26.098982	Up	Let-7
0.0145769	2.9791255	Up	miR-105
0.0105231	14.068628	Up	miR-22
0.0117505	3.9186301	Up	miR-34b
0.0056209	22.5594094	Down	miR-15
0.0002857	15.966217	Down	miR-141
0.0015963	28.210505	Down	miR-346

**Table 2:** Correlation between miR-346 expression and clinicopathological information of HCC patients (n=110)

Characteristics	All patients	miR-346	miR-346	p Value chi-squared test
		low expression^a^	high expression^a^	
**Total case**	110	55	55	
**Age(years)**				
<60	83	42	41	0.825
≥60	27	13	14	
**Gender**				
Male	80	37	43	0.284
Female	30	18	12	
**HbeAg**				
Negative	2	1	1	1.000
Positive	108	54	54	
**Cirrhosis**				
Absent	32	13	19	0.294
Present	78	42	36	
**ALT(U/L)**				
≤45	35	14	21	0.219
>45	75	41	34	
AFP(ng/ml)				
≤13.6	26	10	16	0.262
>13.6	84	45	39	
**Tumor size(cm)**				
≤5	33	10	23	0.012*
>5	77	45	32	
**Vascular invasion**				
Absent	67	29	38	0.118
Present	43	26	17	
**TNM grade**				
I+II	78	32	46	0.006*
III+IV	32	23	9	

^a^The median expression level of miR-346 was used as the cutoff

*Indicates p value<0.05

### MiR-346 suppressed proliferation *in vitro* was associated with cell cycle

**Figure 3:**
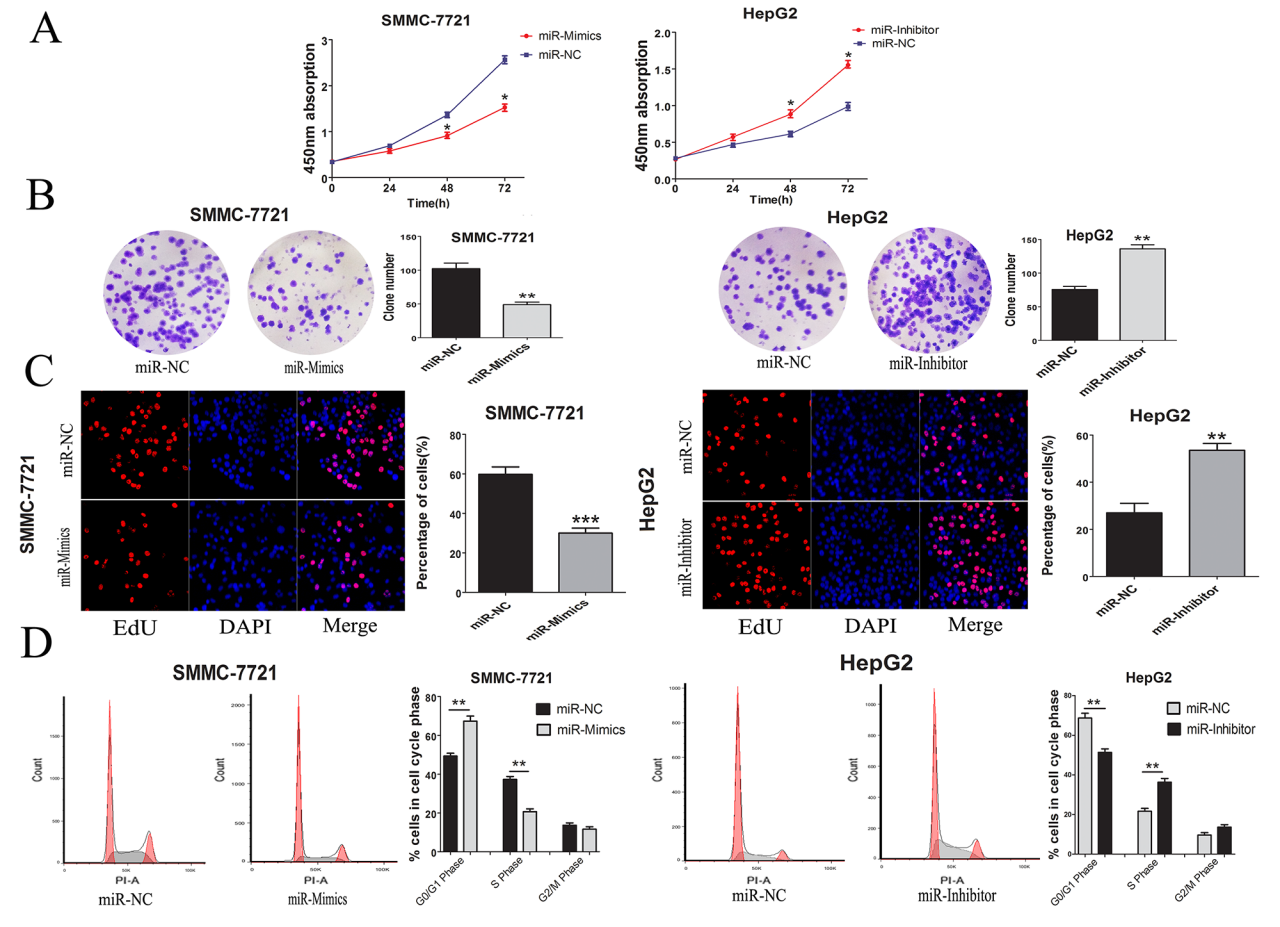
MiR-346 suppressed proliferation *in vitro* was associated with cell cycle **(A)** Proliferation ability was detected by CCK8 assay, over-expression of miR-346 inhibited SMMC-7721 cells proliferation, whereas knock-down of miR-346 promoted HepG2 cells proliferation. **(B)** Colony formation assays were performed on differently treated HCC cells for two weeks, representative graphs are shown. **(C)** EdU immunofluorescence staining confirmed the function of miR-346 on HCC cells proliferation. Original magnification was 200×. Stable over-expression of miR-346 decreased the proliferation of SMMC-7721 cells while knockdown of miR-346 increased the proliferation of HepG2 cells. **(D)** Cell-cycle analysis of SMMC-7721 cells stable over-expressing miR-346 and HepG2 cells stable silenced miR-346 expression was conducted by flow cytometry. The distribution of the cell cycle was shown in the graphs. All experiments were performed in triplicate and presented as the mean ± SEM. (*P<0.05, **P<0.01, ***P<0.0001).

Based on the correlations between miR-346 expression and tumor size from the Chi-square analyses, CCK-8 assays were conducted to explore the potential function of miR-346 on HCC cells. The results showed over-expression of miR-346 could inhibit the proliferation of SMMC-7721. Interestingly, the HepG2 with low-expression of miR-346 also showed more proliferation capacity compared with the control cells (Figure 3A). To further confirm the results above, a series of functional assays including plate cloning and EdU were performed to test the effects of miR-346 on HCC proliferation. As shown in Figure 3B and 3C, miR-346 indeed displayed the function to prevent the proliferation of HCC cells *in vitro*. Since miR-346 was demonstrated to be involved in the modulation of cell proliferation, we next analyzed whether the loss or gain of miR-346 can affect the cell cycle of HCC. The results of cell cycle showed the HCC cells treated with miR-346 mimics, inhibitor and negative control exerted differential distribution of cell cycle phases. The increased percentage of G1 phase was obtained in miR-346 over-expressed HCC cells while the loss of miR-346 could caused the up-regulation of S phase (Figure 3D).

### MiR-346 induced the suppression of SMYD3

**Figure 4:**
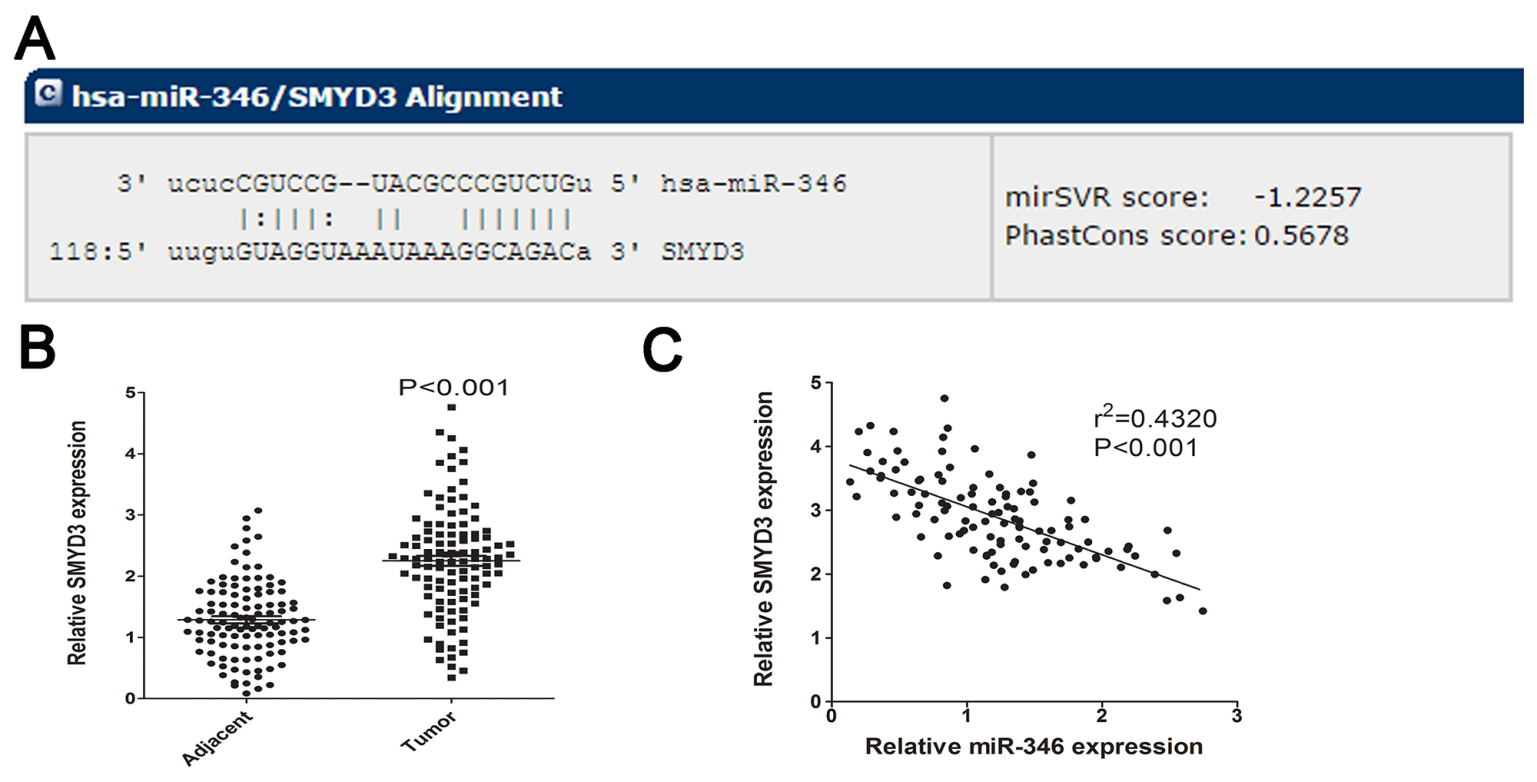
SMYD3 was predicted as target for miR-346 **(A)** Bioinformatic prediction was employed to analyze the potential target gene of miR-346. The detailed sequence information of binding site was presented. **(B)** SMYD3 was significantly increased in HCC tissues compared with the control. **(C)** By utilizing the Pearson correlation analysis, we found miR-346 expression was negatively correlated with the expression of SMYD3 in HCC tissues. Data are presented as means±SEM.

To determine which target gene was responsible for the function of miR-346 on HCC proliferation, we predicted the target gene of miR-346 via utilizing two Bioinformatics databases: Target Scan (http://www.targetscan.org) and miRanda (http://www.microrna.org/). The results of prediction showed the overlapping target gene for miR-346 from these two databases was SMYD3 contained a conserved putative target site for miR-346 (Figure 4A). Then the expression of SMYD3 was found to be obviously up-regulated in HCC tissues compared with the corresponding adjacent tissues (Figure 4B). By using the Pearson correlation analysis, the expression of SMYD3 was found to be negatively associated with miR-346 expression (Figure 4C).

### MiR-346 suppressed proliferation of HCC by targeting SMYD3

**Figure 5:**
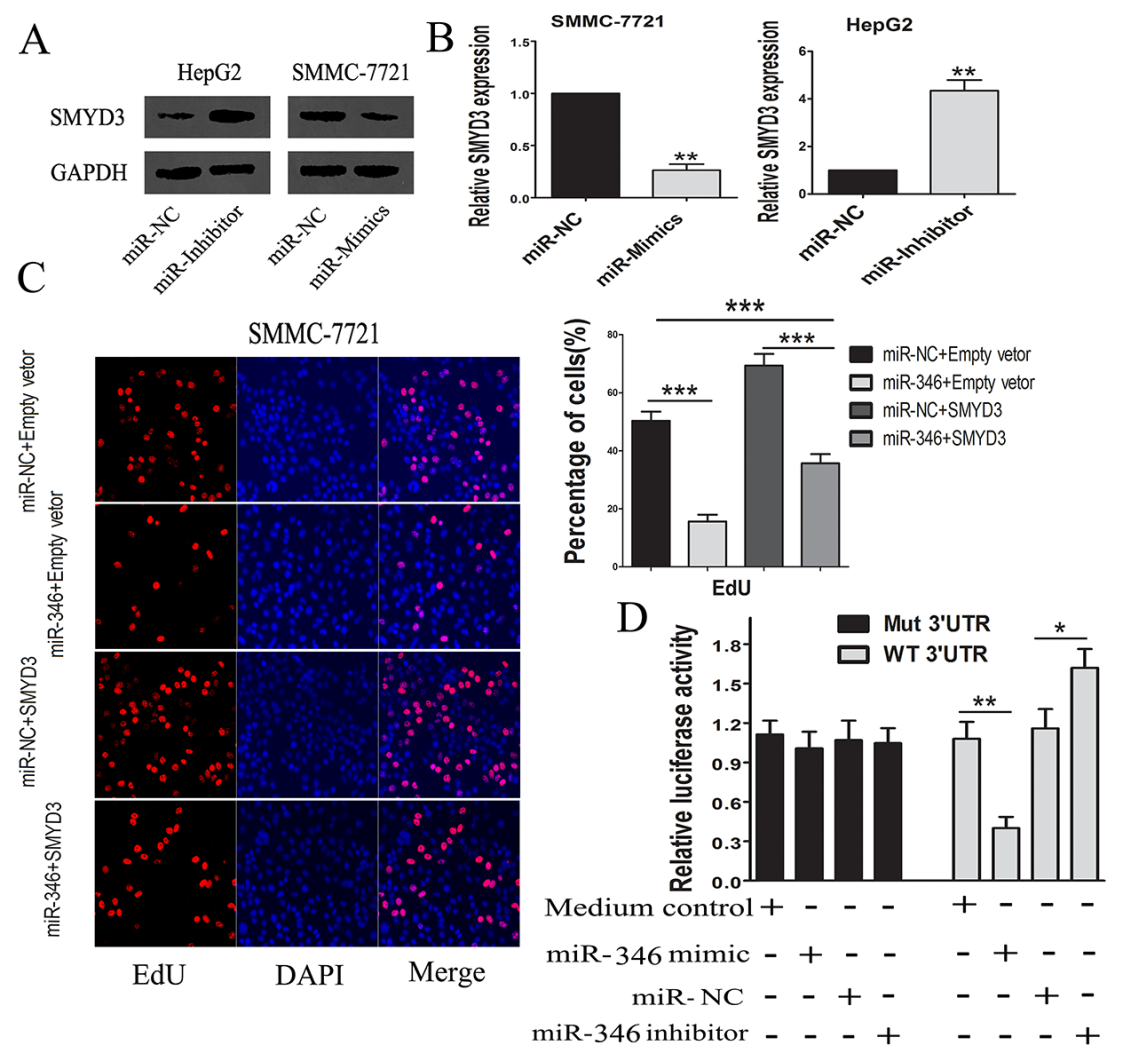
MiR-346 induced the down-regulation of SMYD3 **(A,B)** The findings from the real-time-PCR and western blot suggested that over-expression of miR-346 could suppress the expression of SMYD3 in HCC cells. **(C)** By performing EdU assay, the results showed that up-regulation of SMYD3 could partially neutralize the inhibiting effects of miR-346 on proliferation in SMMC-7721 cells. **(D)** MiR-346 mimic significantly suppressed luciferase activity of SMYD3 containing a wild-type 3′-UTR, but showed no effect on activity of SMYD3 with a mutant 3′-UTR, meanwhile treatment with miR-46 inhibitor increased luciferase activity of SMYD3. Collectively, the results above indicated SMYD3 was a direct target gene of miR-346. Data are presented as means±SEM. (*P < 0.05, **P < 0.01, ***P < 0.0001).

We next detected whether the expression of SMYD3 could be regulated by miR-346. The real-time PCR and western blot confirmed that the miR-346 could inhibit the SMYD3 expression in HCC cells (Figure 5A and 5B). To examine the effects of SMYD3 underlying the inhibitory effects of miR-346 on HCC cells, we transfected the miR-346 over-expressed SMMC-7721 cells with SMYD3-expressing lentivirus. Via performing the EdU assays, we found that over-expression of SMYD3 partially counteracted the inhibitory effects of miR-346 on HCC proliferation (Figure 5C). The results of the EdU assays proved that miR-346 prevented HCC proliferation partially through suppressing the expression of SMYD3. Our data illuminated that miR-346 was a significant upstream molecule which modulated the expression of SMYD3. We also constructed luciferase vectors carrying the 3’-UTR of SMYD3 behind the firefly luciferase gene coding region. As indicated in Figure 5D, miR-346 mimics significantly suppressed luciferase activity of SMYD3 containing a wild-type 3’-UTR, but had no effect on activity of SMYD3 with a mutant 3’-UTR, meanwhile treatment with miR-346 inhibitor increased luciferase activity of SMYD3.

### MiR-346 inhibited tumor growth *in vivo*

**Figure 6:**
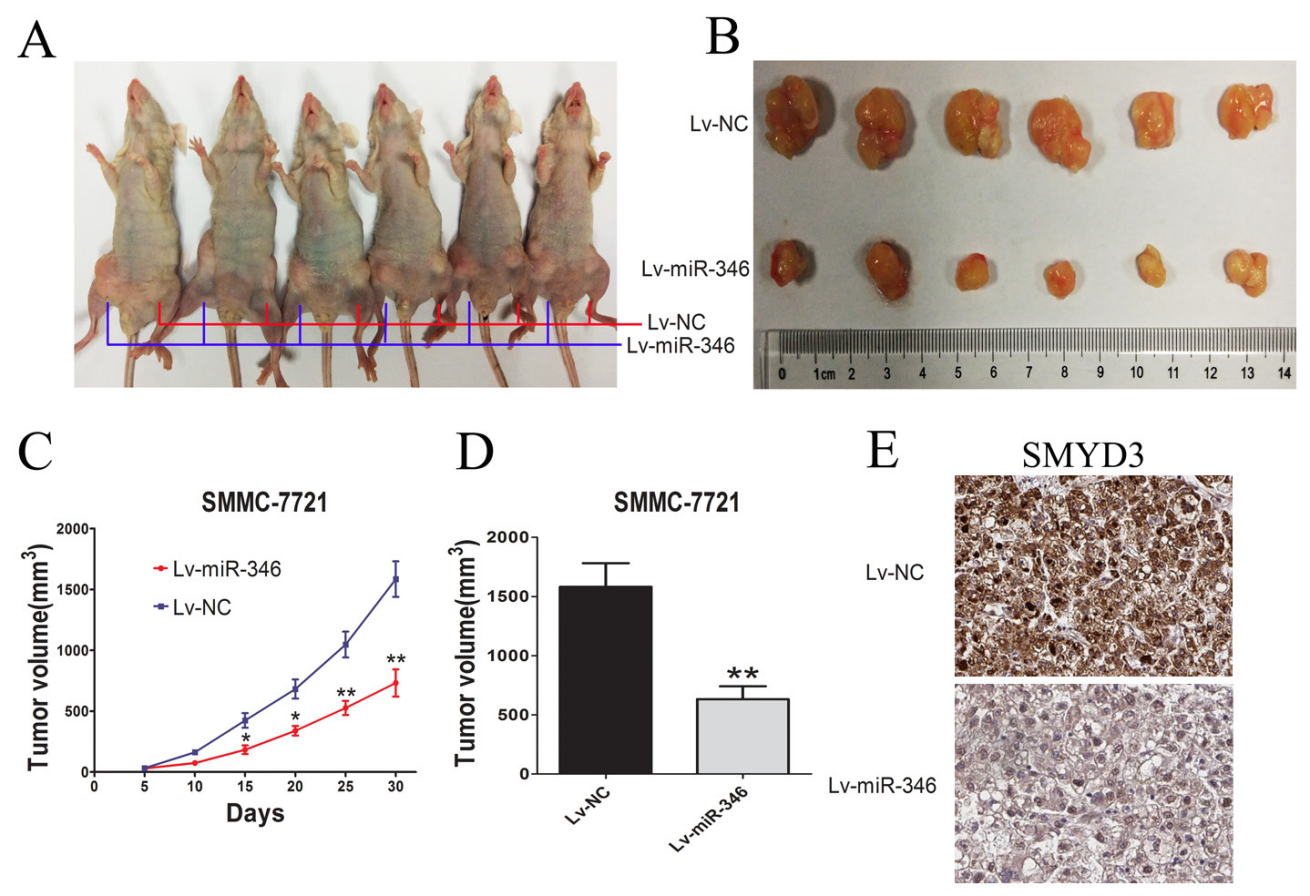
MiR-346 inhibited tumor growth *in vivo* **(A,B)** BALB/c Nude mice (6 weeks of age) were subcutaneous transplantated with SMMC-7721 cells, Lv-miR-346 SMMC-7721 cells(1×10^7^) in the right groin and Lv-NC SMMC-7721 (1×10^7^) cells in the left groin. **(C,D)** The volume of tumors was calculated every 5 days after transplantation and mice were sacrificed 30 days after implantation. MiR-346 decreased the tumor growth of SMMC-7721 cells in nude mice. The volume of each tumor was calculated as the length × width^2^ × 0.5. **(E)** SMYD3 expression level in the tumor samples determined by IHC. Original magnification 400×.

To further examine the effects of miR-346 on tumorigenesis *in vivo*, nude mice were subcutaneously injected with SMMC-7721 cells stably transfected with the ectopic expression lentivirus of miR-346 (Figure 6A). The tumor size was monitored for about every 5 days after administration. As shown in Figure 6B-6D, tumor growth was dramatically suppressed by the miR-346 from day 5 to till sacrifice. These findings strongly supported the viewpoint that miR-346 could inhibit the proliferation of HCC. We also detected the expression of SMYD3 in tumor section by IHC. As presented in Figure 6E, we found that the expression of SMYD3 was suppressed in the subcutaneous tumor.

### MiR-346 was associated with prognosis of HCC patients

**Figure 7:**
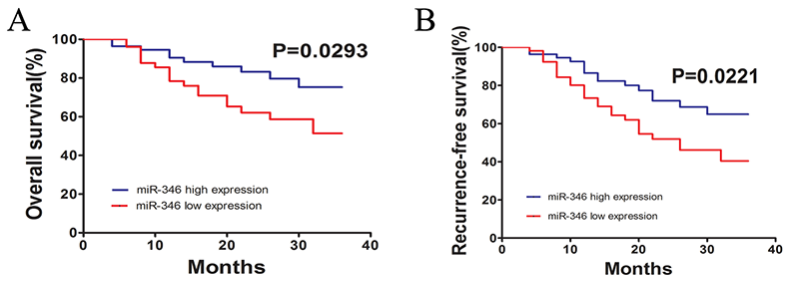
miR-346 was significantly associated with prognosis of HCC **(A,B)** The findings of Kaplan–Meier analysis indicated that patients with low expression of miR-346 had a better OS and RFS than the miR-346 high expression group. The difference was statistically significant.

At last, we performed Kaplan–Meier analysis to investigate the correlation between the miR-346 expression and overall survival (OS) or relapse-free survival (RFS) of the HCC patients (Figure 7A and 7B). Used median as cutoff, we divided 110 HCC samples into two subgroups including miR-346 high group and low group. And the results of Kaplan–Meier analysis showed that HCC patients with low expression of miR-346 had a better OS and RFS compared with the miR-346 high expression group. Then the results of Cox proportional regression analysis also demonstrated that miR-346 might be a prognostic factor for the HCC patients (adjusted hazard ratio (AHR): 0.116 [95% Confidence Interval (CI):0.033–0.445]) (Table 3).

**Table 3:** Cox proportional hazards analyses

Clinical factor	p Value	Univariable analysis		p Value	Multivariable analysis	
		HR	95%CI		AHR	95%CI
**MiR-346**	0.037*	0.236	0.112-0.501	0.003*	0.116	0.033-0.445
**Age**	0.329	1.349	0.740-2.459	0.221	1.252	0.725-2.162
**Gender**	0.228	0.641	0.311-1.321	0.211	1.422	0.819-2.696
**TNM**	0.000	12.394	3.703-41.486	0.000	18.549	4.489-26.269
**AFP**	0.237	1.472	0.776-2.795	0.101	0.625	0.357-1.095
**HBV/HCV**	0.491	1.226	0.694-2.168	0.886	0.961	0.557-1.657
**VI**	0.001	5.615	1.079-12.114	0.000*	11.819	9.063-12.531

VI: vascular invasion; HR: hazard ratio; AHR: adjusted hazard ratio; CI: confidence interval. *Indicates p <0.05.

## DISCUSSION

Previous studies have demonstrated HCC to be one of the most lethal forms of cancer worldwide. HCC patients have high malignancy and poor prognosis, especially in patients who fail first-line systemic therapy. Therefore, it is very essential to identify new diagnostic and prognostic makers for HCC patients. Increasing evidences have showed several pathogen-associated molecular and signaling pathways are involved in the carcinogenesis of HCC. Additionally, miRNA, approximately 19-25 nucleotides in length, can act as either tumor suppressors or oncogenes via targeting anti-oncogenes or oncogenes. Several miRNA have been identified to display a critical role in regulating HCC tumorigenesis and the underlying signaling networks [20, 21]. Illuminating the underlying mechanisms of miRNA in pathogenesis of liver cancer can provide diagnostic and therapeutic strategies for the patients with HCC. MiR-346 is previously identified as closely related to the development and progress of various malignancies including squamous cell carcinoma, cervical cancer, prostate cancer, non-small cell lung cancer and haryngeal carcinoma [22-25]. However, the clinical significance of miR-346 in HCC and the underlying molecular mechanisms still require elusive.

In our current study, we focused on the miR-346 expression in HCC tissues and its effects on the malignant phenotype of HCC cells including proliferation and cell cycle. The results showed that miR-346 expression was significantly decreased in HCC tumor tissues compared with the corresponding adjacent non-tumorous tissues. Besides, using HCC cells and human normal L02 liver cells, we confirmed that miR-346 expression was down-regulated in the HCC cells in comparison with L02 cells. The results above indicated that the aberrant expression of miR-346 has the potential for the early detection HCC. In addition, the results of *Chi-square* analysis showed the expression of miR-346 associated with the tumor size and TNM of HCC patients. To further confirm the potential correlations between the expression of miR-346 and tumor size, a series of functional assays including CCK-8, plate cloning and EdU were performed to determine whether the gain or loss of miR-346 could affect the HCC proliferation. The results we achieved suggested miR-346 could suppress the HCC proliferation *in vitro*. Besides, we also found the inhibitory effects of miR-346 on proliferation were associated with cell cycle. Furthermore, we confirmed miR-346 could prevent the HCC proliferation *in vivo*. Taken together, we demonstrated that miR-346 was a potential tumor suppressor of HCC.

The results above have preliminarily confirmed miR-346 could display a crucial role in the carcinogenesis of HCC, however, the precise mechanisms underlying its effects were still unknown. By utilizing the bioinformatics prediction programs, we found that the 3’-UTR of SMYD3 contained a conserved putative target site for miR-346. Interestingly, previous studies have reported that SMYD3 was a leading gene relating to HCC proliferation. Specifically speaking, SMYD3 was demonstrated to be a protein methyltransferase implicated in cancer development. It was reported that the suppression of SMYD3 could attenuated malignant phenotype of prostate cancer either by deleting the SET function domain or silencing SMYD3, indicating the function of SMYD3 on tumor growth might associated with histone methyltransferase activity. In addition, SMYD3 could regulated the CCND2 through the transcriptional regulation such as trimethylation of H4K20 [19]. SMYD3 was also co-located with H2A.Z.1K101me2 at the promote region of cyclin A1, resulting in the transcriptional activation [26]. Coincidentally, the effects of SMYD3 on HCC cells seemed to associate with the abnormal expression of miR-346. Via using real-time-PCR, we confirmed the expression of SMYD3 was obviously up-regulated in the HCC tissues. And the results of Pearson correlation analyses showed that miR-346 expression was negatively associated with the expression of SMYD3, which indicated miR-346 was a crucial upstream regulatory molecule of SMYD3[17, 27, 28]. Therefore, by using western blot and real-time-PCR, we identified the expression of miR-346 was over-expression in HCC cells transfected with miR-346 inhibitor and down-regulated in HCC cells treated with miR-346 mimics compared with the control. What’s more, the results of luciferase reporter assay suggested that SMYD3 was a direct target gene of miR-346. To further examine the direct interaction between SMYD3 and miR-346, we up-regulated the SMYD3 expression in SMMC-7721 with up-regulation of miR-346 and performed the EdU proliferation assays. The results showed inhibitory effects of miR-346 on HCC proliferation can be partially neutralized by the over-expression of SMYD3, which implied SMYD3 was an oncogene in HCC as a target of miR-346. Furthermore, Kaplan–Meier analyses and Cox proportional regression analyses demonstrated miR-346 as an independent prognostic factor for the HCC patients. In addition, the miR-346 was previously identified as an oncogenic function in human cancers such as breast cancer, lung cancer and nasopharyngeal carcinoma, etc. Here, in this study, we obtained the down-regulated miR-346 in HCC tumor tissues. Further investigation revealed that miR-346 was involved in the development of HCC by acting as a tumor suppresser gene. As we known, a certain miRNA might act as bidirectional function in different cancers. For example, miR-141 could promote prostate cancer cell proliferation through inhibiting KLF9 expression while it can also inhibit vascular smooth muscle cell proliferation through targeting PAPP-A or ZEB2 to suppress HCC progression [29-31]. The different function of certain miRNA might associated with different human disease as well as the endogenous expression of miRNA in different human organs. We will investigate the detailed mechanism of the suppression of miR-346 in human HCC in further study.

In conclusion, we illuminate that miR-346 act as a tumor suppressor miRNA in HCC tumorigenesis and progression. Furthermore, miR-346 prevents HCC proliferation partially through modulating the expression of SMYD3. Besides, Kaplan–Meier analyses and Cox proportional regression analyses suggest that miR-346 is an independent prognostic factor for the patients with HCC. Taken together, all findings in this study are in favor of understanding the mechanisms of miRNA function in HCC carcinogenesis and advancement which can enable us to explore novel prognosis markers and potential targets for cancer therapy in the near future.

## MATERIALS AND METHODS

### Patient samples and cell lines

110 paired HCC fresh tissues consist of tumors and adjacent normal samples were obtained from patients who underwent liver resection at the Liver Transplantation Center in The First Affiliate Hospital of Nanjing Medical University between October 2014 and November 2015. All patients provided their written informed consent to participate in this study. The fresh tissue samples which were confirmed by the histopathological examination were collected in the operating room and processed immediately. Each sample was frozen and stored at liquid nitrogen. The HepG2, SNU423, SMMC-7721, Hep3B, 97H, 97L and Huh7 HCC cell lines and the human normal L02 cell line used in this study were obtained from KeyGen (Nanjing KeyGen Biotech Co.Ltd, China). All of the cells were cultured in DMEM medium(GIBCO, Carlsbad, USA) pre-treated with 10% fetal bovine serum, 80 U/ml of penicillin sodium at 37°C in humidified air containing 5% carbon dioxide.

### Quantitative RT-PCR

Trizol reagent (Sigma) was used to isolate the total RNA from tissues and cells. Expression of miR-346 was determined using MicroRNA First-Strand Synthesis and miRNA Quantitation kits (Takara, Dalian, China) according to the manufacturer’s instructions. The Ct values of U6 and GAPDH were used as the internal control to normalize the relative expression of miR-346 and SMYD3, respectively.

### Cell transfection

Firstly, HCC cells (1×10^5^) were implanted in 6-well plates. One day after the implantation, the cells were transfected with 25 nM of miR-346 mimics, anti-miR-346 inhibitor and the negative control (GenePharma, Shanghai, China), using Lipofectamine 2000 reagent (GIBCO, Carlsbad, USA) according to the manufacturer’s protocol. Meanwhile, all miR-346 and SMYD3 ectopic expression lentivirus as well as the negative control lentivirus were purchased from Gene Chem (Shanghai, China). All lentiviral vectors expressed enhanced green fluorescent protein (GFP), the expression of miR-346 and SMYD3 in the treated cells was confirmed by real-time-PCR 48h after the transfection.

### Cell proliferation assay

The proliferation ability of HCC cells was tested by clone formation assay, cell counting kit-8 (Dojindo Laboratories, Kumamoto, Japan), The EDU (5-ethynyl-2′-deoxyuridine) immunofluorescence staining assay (Millipore, MA) according to the manufacturer’s instructions.

### Flow cytometry analysis of cell cycle

For cell-cycle analysis, cells were subjected to serum starvation to induce cell cycle synchronization. The cells at the logarithm growth period were harvested and fixed in 70% ethanol for a night at -20°C. The next day, the cells were washed and incubated in propidium iodide (PI) (Multi Science) and analyzed by flow cytometry.

### *In vivo* experiments

BALB/c Nude mice, 6 weeks of age or older, were purchased from the animal center of Nanjing University (Nanjing, China), raised and permitted by the Nanjing medical University animal studies committee. In the subcutaneous transplantation model, 6 mice was implanted with Lv-miR-346-SMMC-7721 cells(1×10^7^) in the right groin, and Lv-NC-SMMC-7721 cells (1×10^7^) in the left groin. We calculated the volume of tumors every 5 days after transplantation and sacrificed 30 days after implantation.

### Target prediction for microRNAs

Multiple database was employed to predict the potential targets for miRNAs. The Bio-informatics programme including TargetScan (http://www.targetscan.org/), miRanda (http://www.microrna.org/) as well as the PicTar (pictar.mdc-berlin.de/) was used. Potential targets was screened by the rank score provided by different database.

### Luciferase assay

DNA sequences containing the miR-346 binding site on the 3’-UTR of SMYD3 were cloned into the downstream of the firefly luciferase stop codon in a pmirGLO control vector (Promega, Milan, Italy). SMMC-7721 cells were implanted in 24 well plates. The following day, the cells were respectively co-transfected with 25 nM of miR-346 mimic, miR-346 inhibitor or miR-NC (GenePharma, Shanghai, China) and 500 ng of 3’-UTR (WT or Mut) of SMYD3 pmirGLO recombinant vectors. Then the cells were harvested 24h after transfection. Firefly luciferase activity was detected with Dual Luciferase Assay (Promega, Milan, Italy) in accordance with the manufacturer’s instructions.

### Immunohistochemical assay

The tissue samples were fixed in 4% paraformaldehyde at 4°C and sectioned into slices. After deparaffinage and rehydration, the sections were put into pressure cooker for 5 minutes to restore the antigen in the nuclear by using citrate method. H2O2 suppresses endogenous peroxidase activity to reduce background. Blocked in normal goat serum with 5% BSA in TBS for 1 hour at room temperature was also needed. The sections were incubated with primary antibody (1:400 dilution) overnight at 4°C and then washed in PBS for three times. After incubated with secondary antibodies, sections were subjected to DAB reaction. Photograph the sections by using a digitalized microscope camera (Nikon, Tokyo, Japan).

### Western blotting

The RIPA buffer with phenylmethanesulfonylfluoride (Beyotime, Nantong, China) was applied to extract the proteins from tissues or cells. Total amount of 100ng protein loading in each lane was determined by normalized with GAPDH. The results were analyzed by Pierce ECL Substrate Western blot detection system (Thermo Scientific, IL, USA).

### Statistical analysis

All experimental assays were performed independently in triplicate. Two-tailed student t test was used to assess the statistical differences between groups. All statistical data were carried out using Statistical Program for Social Sciences 18.0 software (SPSS, USA) and presented with Graphpad prism 5.0 (GraphPad Software, CA). P value less than 0.05 was considered as significant.

## References

[1] Giordano S, Columbano A (2014). Met as a therapeutic target in HCC: facts and hopes. J Hepatol.

[2] Greten TF, Wang XW, Korangy F (2015). Current concepts of immune based treatments for patients with HCC: from basic science to novel treatment approaches. Gut.

[3] Rossi JJ (2009). New hope for a microRNA therapy for liver cancer. Cell.

[4] Mori M, Triboulet R, Mohseni M, Schlegelmilch K, Shrestha K, Camargo FD, Gregory RI (2014). Hippo signaling regulates microprocessor and links cell-density-dependent miRNA biogenesis to cancer. Cell.

[5] Rubio-Somoza I, Weigel D, Franco-Zorilla JM, Garcia JA, Paz-Ares J (2011). ceRNAs: miRNA target mimic mimics. Cell.

[6] Ventura A, Young AG, Winslow MM, Lintault L, Meissner A, Erkeland SJ, Newman J, Bronson RT, Crowley D, Stone JR, Jaenisch R, Sharp PA, Jacks T (2008). Targeted deletion reveals essential and overlapping functions of the miR-17 through 92 family of miRNA clusters. Cell.

[7] Voorhoeve PM, le Sage C, Schrier M, Gillis AJ, Stoop H, Nagel R, Liu YP, van Duijse J, Drost J, Griekspoor A, Zlotorynski E, Yabuta N, De Vita G (2006). A genetic screen implicates miRNA-372 and miRNA-373 as oncogenes in testicular germ cell tumors. Cell.

[8] Pencheva N, Tran H, Buss C, Huh D, Drobnjak M, Busam K, Tavazoie SF (2012). Convergent multi-miRNA targeting of ApoE drives LRP1/LRP8-dependent melanoma metastasis and angiogenesis. Cell.

[9] Shimono Y, Zabala M, Cho RW, Lobo N, Dalerba P, Qian D, Diehn M, Liu H, Panula SP, Chiao E, Dirbas FM, Somlo G (2009). Downregulation of miRNA-200c links breast cancer stem cells with normal stem cells. Cell.

[10] Xu B, Hsu PK, Stark KL, Karayiorgou M, Gogos JA (2013). Derepression of a neuronal inhibitor due to miRNA dysregulation in a schizophrenia-related microdeletion. Cell.

[11] Lee YS, Nakahara K, Pham JW, Kim K, He Z, Sontheimer EJ, Carthew RW (2004). Distinct roles for Drosophila Dicer-1 and Dicer-2 in the siRNA/miRNA silencing pathways. Cell.

[12] Zhao Y, Ransom JF, Li A, Vedantham V, von Drehle M, Muth AN, Tsuchihashi T, McManus MT, Schwartz RJ, Srivastava D (2007). Dysregulation of cardiogenesis, cardiac conduction, and cell cycle in mice lacking miRNA-1-2. Cell.

[13] Giakountis A, Moulos P, Sarris ME, Hatzis P, Talianidis I (2017). Smyd3-associated regulatory pathways in cancer. Semin Cancer Biol.

[14] Kim JM, Kim K, Schmidt T, Punj V, Tucker H, Rice JC, Ulmer TS, An W (2015). Cooperation between SMYD3 and PC4 drives a distinct transcriptional program in cancer cells. Nucleic Acids Res.

[15] Liu C, Wang C, Wang K, Liu L, Shen Q, Yan K, Sun X, Chen J, Liu J, Ren H, Liu H, Xu Z, Hu S (2013). SMYD3 as an oncogenic driver in prostate cancer by stimulation of androgen receptor transcription. J Natl Cancer Inst.

[16] Mazur PK, Reynoird N, Khatri P, Jansen PW, Wilkinson AW, Liu S, Barbash O, Van Aller GS, Huddleston M, Dhanak D, Tummino PJ, Kruger RG, Garcia BA (2014). SMYD3 links lysine methylation of MAP3K2 to Ras-driven cancer. Nature.

[17] Sarris ME, Moulos P, Haroniti A, Giakountis A, Talianidis I (2016). Smyd3 Is a Transcriptional Potentiator of Multiple Cancer-Promoting Genes and Required for Liver and Colon Cancer Development. Cancer Cell.

[18] Sponziello M, Durante C, Boichard A, Dima M, Puppin C, Verrienti A, Tamburrano G, Di Rocco G, Redler A, Lacroix L, Bidart JM, Schlumberger M, Damante G (2014). Epigenetic-related gene expression profile in medullary thyroid cancer revealed the overexpression of the histone methyltransferases EZH2 and SMYD3 in aggressive tumours. Mol Cell Endocrinol.

[19] Vieira FQ, Costa-Pinheiro P, Almeida-Rios D, Graca I, Monteiro-Reis S, Simoes-Sousa S, Carneiro I, Sousa EJ, Godinho MI, Baltazar F, Henrique R, Jeronimo C (2015). SMYD3 contributes to a more aggressive phenotype of prostate cancer and targets Cyclin D2 through H4K20me3. Oncotarget.

[20] Cochetti G, Poli G, Guelfi G, Boni A, Egidi MG, Mearini E (2016). Different levels of serum microRNAs in prostate cancer and benign prostatic hyperplasia: evaluation of potential diagnostic and prognostic role. Onco Targets Ther.

[21] Du L, Borkowski R, Zhao Z, Ma X, Yu X, Xie XJ, Pertsemlidis A (2013). A high-throughput screen identifies miRNA inhibitors regulating lung cancer cell survival and response to paclitaxel. RNA Biol.

[22] Guo J, Lv J, Liu M, Tang H (2015). miR-346 Up-regulates Argonaute 2 (AGO2) Protein expression to augment the activity of other microRNAs (miRNAs) and contributes to cervical cancer cell malignancy. J Biol Chem.

[23] Yang F, Luo LJ, Zhang L, Wang DD, Yang SJ, Ding L, Li J, Chen D, Ma R, Wu JZ, Tang JH (2017). MiR-346 promotes the biological function of breast cancer cells by targeting SRCIN1 and reduces chemosensitivity to docetaxel. Gene.

[24] Chen B, Pan W, Lin X, Hu Z, Jin Y, Chen H, Ma G, Qiu Y, Chang L, Hua C, Zou Y, Gao Y, Ying H, Lv D (2016). MicroRNA-346 functions as an oncogene in cutaneous squamous cell carcinoma. Tumour Biol.

[25] Chen Y, Du J, Zhang Z, Liu T, Shi Y, Ge X, Li YC (2014). MicroRNA-346 mediates tumor necrosis factor alpha-induced downregulation of gut epithelial vitamin D receptor in inflammatory bowel diseases. Inflamm Bowel Dis.

[26] Tsai CH, Chen YJ, Yu CJ, Tzeng SR, Wu IC, Kuo WH, Lin MC, Chan NL, Wu KJ, Teng SC (2016). SMYD3-mediated H2A.Z.1 methylation promotes cell cycle and cancer proliferation. Cancer Res.

[27] Luo XG, Zhang CL, Zhao WW, Liu ZP, Liu L, Mu A, Guo S, Wang N, Zhou H, Zhang TC (2014). Histone methyltransferase SMYD3 promotes MRTF-A-mediated transactivation of MYL9 and migration of MCF-7 breast cancer cells. Cancer Lett.

[28] Luo XG, Zou JN, Wang SZ, Zhang TC, Xi T (2010). Novobiocin decreases SMYD3 expression and inhibits the migration of MDA-MB-231 human breast cancer cells. IUBMB Life.

[29] Zhang Y, Chen B, Ming L, Qin H, Zheng L, Yue Z, Cheng Z, Wang Y, Zhang D, Liu C, Bin W, Hao Q, Song F, Ji B (2015). MicroRNA-141 inhibits vascular smooth muscle cell proliferation through targeting PAPP-A. Int J Clin Exp Pathol.

[30] Wu SM, Ai HW, Zhang DY, Han XQ, Pan Q, Luo FL, Zhang XL (2014). MiR-141 targets ZEB2 to suppress HCC progression. Tumour Biol.

[31] Li JZ, Li J, Wang HQ, Li X, Wen B, Wang YJ (2017). MiR-141-3p promotes prostate cancer cell proliferation through inhibiting kruppel-like factor-9 expression. Biochem Biophys Res Commun.

